# Grounded accountability in life-and-death high-consequence healthcare settings

**DOI:** 10.1108/JHOM-03-2021-0116

**Published:** 2021-08-24

**Authors:** Maureen Alice Flynn, Niamh M. Brennan

**Affiliations:** Health Services Executive , Dublin, Ireland; University College Dublin , Dublin, Ireland

**Keywords:** Accountability, Hospital, Clinical governance, Life-and-death, High-consequence

## Abstract

**Purpose:**

The paper examines interviewee insights into accountability for clinical governance in high-consequence, life-and-death hospital settings. The analysis draws on the distinction between formal “imposed accountability” and front-line “felt accountability”. From these insights, the paper introduces an emergent concept, “grounded accountability”.

**Design/methodology/approach:**

Interviews are conducted with 41 clinicians, managers and governors in two large academic hospitals. The authors ask interviewees to recall a critical clinical incident as a focus for elucidating their experiences of and observation on the practice of accountability.

**Findings:**

Accountability emerges from the front-line, on-the-ground. Together, clinicians, managers and governors co-construct accountability. Less attention is paid to cost, blame, legal processes or personal reputation. Money and other accountability assumptions in business do not always apply in a hospital setting.

**Originality/value:**

The authors propose the concept of co-constructed “grounded accountability” comprising interrelationships between the concept’s three constituent themes of front-line staff’s felt accountability, along with grounded engagement by managers/governors, supported by a culture of openness.

## Introduction

In a healthcare setting, clinical governance is a central mechanism for patient safety ( Scally and Donaldson, 1998 ). Accountability is also at the heart of clinical governance ( Brennan and Flynn, 2013 , p. 119). Unlike bottom-line (for-profit) corporate settings, hospitals’ life-and-death context means that the quality and safety of services is the priority. Traditional hierarchical conceptions of accountability come from bottom-line (for-profit) corporate settings. However, the life-and-death context in hospitals may require a different approach. Healthcare is a high-risk, high-consequence service ( McCall and Pruchnicki, 2017 ; McCarthy *et al.* , 1997 ; Patterson *et al.* , 2004 ). Effective systems of accountability for clinical governance are critical to minimise and control risk. Our research is motivated by a desire to increase understanding of how clinicians, managers and governors enact accountability practices for clinical governance within hospitals.

We interview 41 clinicians, managers and governors in two large academic hospitals who share their experiences responding to critical clinical incidents (where unintended mistakes or harm happen). Twenty-eight (68%) of our 41 interviewees are clinicians; 13 (32%) are administrators. We use the interview data to reveal the practice of accountability for clinical governance, which we develop through a conceptual framework of our emergent concept, “grounded accountability” [1] . Grounded accountability involves clinicians, managers and governors co-constructing accountability through front-line staff’s felt accountability, grounded engagement by managers and governors, supported by a culture of openness.

The complexity of accountability in a life-and-death setting differentiates clinical accountability from hierarchical corporate accountability. We reveal the emergent concept of “grounded accountability”, which reflects interviewee experience of accountability practices. We uncover “felt accountability” at the front line, accommodated by managers’ and governors’ “grounded engagement”. Managers and governors co-construct accountability with front-line staff, supported by a culture of openness. We believe our “grounded accountability” concept has wide applicability beyond the context of our study.

We make three contributions to the literature: (1) we extend the theoretical conception of corporate accountability ( Roberts, 1991 , 2009 ; Roberts and Scapens, 1985 ) and Non-Government Organisation (NGO) accountability ( O'Dwyer and Boomsma, 2015 ) to a new context, critical clinical incidents in a hospital setting; (2) we provide insights from an extreme high-consequence context where accountability practices operate in a life-and-death context and (3) we reveal the emergent concept of “grounded accountability”.

## Literature review and theoretical perspectives

### What is clinical governance?

The United Kingdom (UK) Department of Health (1998) and Scally and Donaldson (1998 , p. 61) first described clinical governance as (emphasis added): “a system through which NHS organisations are *accountable* for continuously improving the quality of their services and safeguarding high standards of care by creating an environment in which excellence in clinical care will flourish”. Clinical governance has parallels with the more widely known corporate governance ( Braithwaite and Travaglia, 2008 ). Clinical governance addresses the structures, systems and processes that assure the quality, accountability and proper management of an organisation’s operation and service delivery ( Bismark *et al.* , 2013 ; Franks, 2001 ; Starey, 2001 ). Achieving effective clinical governance requires a collaborative effort between clinicians, managers and governors, including clarity about their separate and distinct roles ( Flynn and Brennan, 2021 ; Veenstra *et al.* , 2017 ). Accountability is at the heart of clinical governance ( Allen, 2000 ).

### What is accountability?

Accountability requires people to explain and take responsibility for their actions, described as “the giving and demanding of reasons for conduct” ( Roberts and Scapens, 1985 , p. 447). The business literature conceives accountability as retrospective. This conception has implications for healthcare, where there is a need to balance retrospection with a prospective approach focused on anticipating and minimising harm ( Lloyd *et al.* , 2007 ). Transferring accountability practices from a business context to a high-consequence healthcare setting is not straightforward ( Roberts, 1991 , 2009 ). The complexity of accountability in a life-and-death setting differentiates clinical accountability from corporate accountability. When we talk about accountability for clinical governance, we envisage that clinicians, managers and governors explain and take responsibility for safeguarding high standards of care by creating an environment in which excellence in clinical care flourishes.

The constant giving and demanding of reasons for [and results of] conduct can instil a sense of anxiety or vulnerability amongst accountable persons, as they continually strive to demonstrate performance ( O'Dwyer and Unerman, 2008 ; Roberts, 2001 ). Surveillance and audit may reduce individuals’ commitment and loyalty to their organisations and may damage rather than support trust ( O'Neill, 2004 ). Hierarchical forms of accountability can damage the potential to embrace failure as an opportunity for learning ( Roberts, 2001 ). Accountability may place a burden on those who are “expected to provide a convincing account even in situations where this is extremely difficult or even impossible” ( Messner, 2009 , p. 919). Consequently, practitioners do not always meet demands for accountability. Hospitals provide health services to people with varied profiles, where the complexity of service provision makes performance and causality more complex to monitor and evaluate.

### Accountability in a high-consequence healthcare context

Work environments that can threaten the health or lives of people working within the system are known as high-consequence work systems ( McCarthy *et al.* , 1997 ). The probability of harm (adverse events) in healthcare is significant and lies between 3% and 17% internationally ( Brennan *et al.* , 1991 ; Rafter *et al.* , 2017 ; Vincent, 2011 ). In non-healthcare environments, establishing cause and effect between accident and injury is often reasonably straightforward (e.g. speed and road deaths). In contrast, “patients are generally, though not always, sick and separating the harm due to healthcare from that due to illness is often difficult” ( Vincent, 2011 ). Minimising harm is compounded by the complexity (density of interactions between different components) of healthcare. Therefore, healthcare can be described as a high-consequence work system.

The concepts of an imposed, a felt or an adaptive accountability underpin three ideal types of Non-Governmental Organisation (NGO) accountability regimes ( O'Dwyer and Boomsma, 2015 ): (1) An imposed accountability regime prioritises formal, coercive, compliance-based forms of accountability that seek to hold people responsible for their actions in a hierarchical manner using mainly quantitative measures; (2) A felt accountability regime privileges individuals’ motivations and their sense of their responsibilities. Felt accountability involves individuals voluntarily taking responsibility for opening themselves to scrutiny and assessing their performance concerning goals aligned to the organisation’s mission; (3) An adaptive accountability regime is a hybrid seeking to align the core features of an imposed and a felt accountability.

Like NGOs, hospitals are often engaged in complicated and continual balancing acts between accountabilities externally imposed and those internally generated. Accountability, requiring an explanation of conduct, misses this intrinsic motivation to offer up an account rather than respond to a demand for an account. Instead, felt accountability comprises a more dynamic, reflexive and internally focused process, with inter-dependence amongst organisational members, a reciprocal sense of responsibility that is collectively generated ( O'Dwyer and Boomsma, 2015 ).

### Mintzberg’s on-the-ground engagement

Mintzberg believes that managers need to understand what is happening on the ground and experience “on-the-ground engagement” ( Mintzberg, 2017a , b ). Managers face a quandary of connecting with the front-line when the act of managing removes managers from what they are managing ( Mintzberg, 2013 ). Mintzberg (2009) advocates on-the-ground management and grounded learning by managers from front-line staff – the people who viscerally know what is going on. He suggests a process of grounded engagement instead of top-down direction, which he calls “communityship” ( Mintzberg, 2017b ). Grounded engagement is based on the judgement exercised by front-line staff, rooted in their experiences, and drawing on their felt accountability ( Mintzberg, 2013 ), with managers connecting to front-line staff’s on-the-ground experience ( Mintzberg, 2017b ). We coin the phrase “grounded accountability” to capture clinicians, managers and governors co-constructing accountability through front-line staff’s felt accountability, grounded engagement by managers and governors, supported by a culture of openness. In their study of six principles emanating from a large academic health system’s board quality committee, Pronovost *et al.* (2018 , p. 3) emphasise the importance of accountability for performance flowing “from board to bedside and from bedside to board”, which sentiment resonates with our paper.

## Research methodology

Our research question is: What are interviewees’ insights into accountability for clinical governance in life-and-death, high-consequence healthcare settings? We use interviewee insights when something goes wrong (critical clinical incident) to illuminate the practice of accountability. In our research, “critical clinical incident” refers to a situation where the event results in the potential for, or actual, serious harm to a patient, which warrants an immediate investigation and response ( Davies *et al.* , 2003 ).

To guide the identification of hospitals to facilitate the research, we develop inclusion criteria as follows: the hospital (1) has an established hospital board, (2) is an acute general hospital and (3) is a large academic university hospital. Seven of the 48 publicly funded hospitals in Ireland fulfil the inclusion criteria. We start by contacting the CEO/Chair of the board of the first two hospitals listed to explore possible participation in the study. When they verbally agree, we follow up with formal written invitations to the chair of the hospital board and to the CEO. Both hospitals have more than 3,000 whole-time-equivalent (WTE) employees.

We explore accountability practices with clinicians, managers and governors from two hospitals in parallel, not because we expect to find contrasts but to control for circumstances that might make a single hospital unrepresentative. We conduct semi-structured interviews with 41 clinicians, managers and governors from the two hospitals [2] . Through the lead author’s senior clinical experience, we obtain unique access to senior staff and clinicians at the front line, which elicits responses of depth not possible for outsiders. Interviewing people holding different roles allows us to obtain deeper insights by reference to roles held. Both hospital CEOs nominate a link person to coordinate introductions and meetings with interviewees. We provide criteria to guide the nominated link person in identifying potential interviewees, understanding that the power in purposeful sampling lies in selecting information-rich cases for study, a core distinguishing element of qualitative inquiry ( Patton, 2002 ). We provide the nominated link person with a list of targeted roles (e.g. chair, CEO, clinical/medical director etc.) and more generic roles based on experiences (e.g. hospital consultant, advanced nurse practitioners etc.) We have no direct involvement in selecting the specific board members or clinicians who participate. The use of a nominated link person was a practical and necessary expediency (access to list of employees, email addresses etc.).

We send each potential interviewee a letter of introduction and research information sheet by email via the nominated link person who possesses their email addresses. We follow up with a hard copy letter of invitation to participate, which we send directly to interviewees in the internal post. We issue 43 invitations to participate via the nominated link person. Of these, 41 people accept, and two clinicians do not respond to the invitation.

We inform all interviewees about the study in advance, so they have an opportunity to consider the information before confirming a decision to participate. We also inform interviewees about how we handle their data and how we remove identifying characteristics from the transcripts. We provide them with contact details and encourage them to contact the researchers with further recollections and any concerns after the interviews. Finally, we provide interviewees with a research consent form in advance of the fieldwork, including the right to withdraw from the study.

The interview guide (available from the authors on request) has four parts: (1) interviewee background, (2) accountability practices, (3) how practitioners enact best practices in the provision of clinical care during critical clinical incidents and (4) interviewee conclusion and reflections. To not influence interviewee responses, we take care not to disclose the phenomenon of interest (the practice of accountability). We assess the interview guide’s effectiveness through a pilot study with four interviewees (not part of the study findings). Table 1 describes clinician, manager and governor interviewees. Twenty-eight (68%) of our 41 interviewees are clinicians; 13 (32%) are administrators. Some of the clinicians also hold management/governor roles. We exercise judgement in classifying multiple role holders into three groups. The clinicians, managers and governors interviewed have considerable healthcare experience, ranging from 5 to 51 years. Twenty-four (59%) have experience working in a wide range of countries outside Ireland.

We ask interviewees to recall a critical clinical incident as a focus for elucidating their experiences of and observations on the practice of accountability. We use the critical-incident technique ( Flanagan, 1954 ) to elucidate interviewees’ experiences and observations of the practice of accountability for clinical governance. The critical incident lens encourages interviewees to speak about their experiences and reveal the practices of accountability, without being conscious that accountability is the focus of the research. Interviews range from 24 to 98 min (lasting, on average, 53 min). In total, we collect over 36 h of interview data (yielding 306,227 words of transcript). The 41 interviewees share their experiences and reflections in 58 critical clinical narratives (23 in Hospital A, 26 in Hospital B and nine while working in other services) of 50 critical clinical incidents (some interviewees speak about the same critical clinical incident). Twenty-six (45%) of the 58 narratives discuss an unexpected death ( *n* = 20). The critical clinical incidents occurred between 2001 and 2016, with the majority taking place more recently between 2015 and 2016. We develop structured summaries for each critical clinical incident. The summaries facilitate the reduction and retention of data and allow reflection and interaction with the evidence ( O'Dwyer, 2004 ).

**Figure 1:**
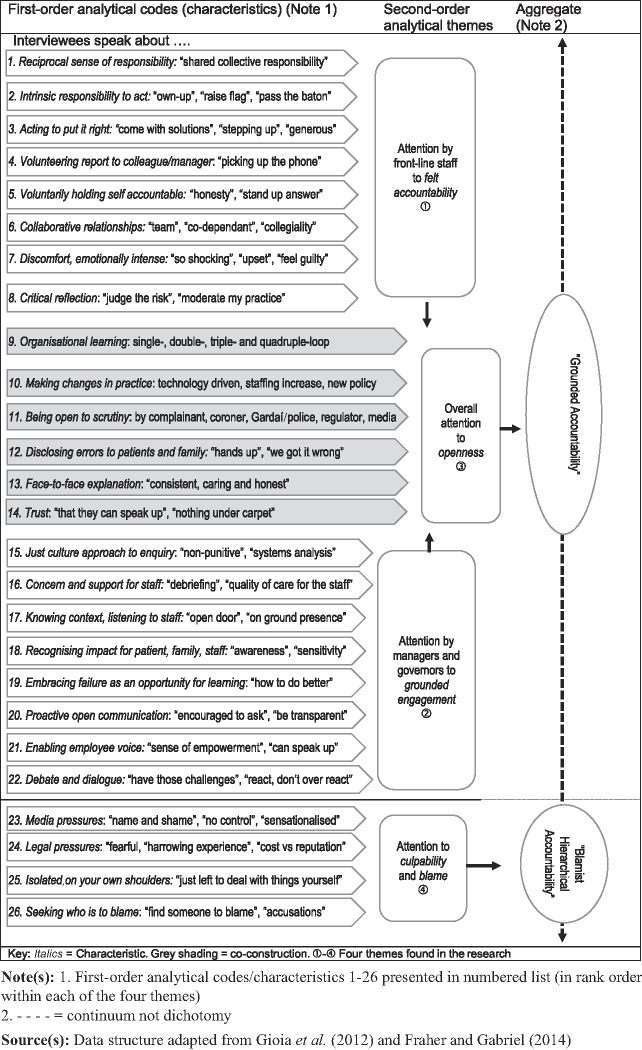
The emergent concept “grounded accountability”

Coding was completed by the first-named author, under the supervision of the second author. Initial topic coding results in 279 codes in 14 groups ( Hennink *et al.* , 2011 ). We then conduct analytical coding by considering the data in context and grouping/re-grouping topic codes into meaningful first-order analytical codes ( Gioia *et al.* , 2012 ). We identify 26 first-order analytical codes (see Figure 1 ). Figure 1 labels the codes and provides key phrases indicating the stem for each characteristic. Four second-order analytical themes emerge. We use the term “attention” across the four second-order analytical theme labels. This abductive approach ( Tavory and Timmermans, 2014 ) allows us to distil the themes into two aggregates which we label “grounded accountability” and “blamist hierarchical accountability”. The dotted arrow between “grounded accountability” and “blamist hierarchical accountability” shows the continuum between both approaches.

The data reveal that “grounded accountability” and a “blamist hierarchical accountability” co-exist to varying degrees. However, “grounded accountability” dominates. “Grounded accountability” emerges strongly and comprises 22 first-order analytical codes/characteristics (a detailed description of the 22 characteristics is available from the authors on request). These 22 first-order analytical codes/characteristics relate to two of the four second-order analytical themes, “attention by front-line staff to felt accountability” and “attention by managers and governors to grounded engagement”. These feed into a third second-order analytical theme, “overall attention to openness”. The “blamist hierarchical accountability” category comprises only 4 of the 26 first-order analytical codes.

## Findings and discussion

This section provides insight into the four main themes emerging from the data (➀ to ➃ in Figure 1 ): attention by front-line staff to felt accountability, attention by managers and governors to grounded engagement, overall attention to openness and attention to culpability and blame. We introduce the emergent concept, grounded accountability, and its 22 characteristics and provide a deeper insight into several characteristics. We also present the practice of accountability for clinical governance revealed in the data.

### Grounded accountability – attention by front-line staff to felt accountability

The first theme, “attention by front-line staff to a felt accountability”, contributes to our “grounded accountability” concept. Our data reveal eight felt accountability characteristics (see Figure 1 , 1–8). Characteristic 1, “Reciprocal sense of responsibility”, is the most frequent characteristic within the felt accountability theme. Interviewees (24) speak about team members’, managers’ and governors’ shared responsibilities for the standard of clinical care provided, suggesting a shared mental model (collectively generated rather than unidirectionally imposed). There is no sense of a silo-style accountability culture ( Pellinen *et al.* , 2018 ). For example, a manager, who is also an executive member of the hospital board, describes a mutual appreciation and acting together to address the challenges in providing safe care for patients in the emergency department (see Quotation 1). The interdependencies of action and the acknowledgement that “one's actions make a difference both to self and others” ( Roberts, 1991 , p. 365) is the basis of this reciprocal sense of responsibility.

Quotation 1: Reciprocal sense of responsibility (Characteristic 1, Figure 1 )

And I think people are starting to appreciate more like, you cannot have a big risk developing in the ED and people are working throughout the organisation to try and remove that risk and thereby working as a team to try and move things along nicely (Source: Manager, Governor).

### Grounded accountability – attention by managers and governors to grounded engagement

The second theme, “attention by managers and governors to grounded engagement”, reveals eight grounded engagement characteristics (see Figure 1 , 15–22). Characteristic 15, “just-culture”, is the most frequent characteristic in the “attention by managers and governors to grounded engagement” theme. Interviewees (35) speak about an enquiry in which front-line staff and others are not punished for actions, omissions or decisions taken by them, which are commensurate with their experience and training, but where gross negligence, wilful violations and destructive acts are not tolerated. In this study, managers and board members describe proactively seeking to move away from a blame approach and encouraging and acknowledging where people speak up – to set this as the norm and the expectation. For example, a member of the team reviewing a critical clinical incident where a patient received an intravenous drug to which they are allergic (the patient was successfully resuscitated and cared for in the intensive care unit for several days) talked about taking a just culture approach (see Quotation 2).

Quotation 2: Just culture approach (i) (Characteristic 15, Figure 1 )

A culture whereby people feel as though you can report incidents and they'll be looked at in a just way but then they'll also be looked at in a way where if somebody is outside of their scope or working in such a way that there's competence issues well then that will be addressed as well (Source: Clinician, Manager).

While the term “just culture” is not often used by interviewees, such a culture’s features are often described (see Quotation 3).

Quotation 3: Just culture approach (ii) (Characteristic 15, Figure 1 )

… I think we have a culture which tries to promote a no-blame perspective and we try and foster a culture of openness and that as I say, while patient care and patient safety is paramount, that equally if things do happen, which should not happen, that people recognise that there's a need to appropriately put that out in the open as opposed to conceal that, so that we can have a better understanding of how it happened. Why it happened. What we can do to address it. And more importantly, depending on the nature of it, that we can put safeguards or controls or systems or whatever in place to ensure that that situation, if it's of a particular magnitude does not happen again. (Source: Manager).

### Grounded accountability – overall attention to openness

The third theme, “overall attention to openness”, stems from the understanding that a simple disclosure or provision of information (accounting) alone does not comprise accountability ( Cooper and Johnson, 2012 ). The data reveals six characteristics of overall openness (see Figure 1 , 9–14). “Organisational learning” is the most frequent characteristic of openness. Wilkinson *et al.* (2004 , p. 112) identify managers’ role to manage possible tensions from “top down imposition of clinical governance and the bottom up development of the learning organisation in such a way as to take advantage of the potential benefits of each”. A clinician illustrates the potential impact on learning by describing the learning and new mental health assessment tool developed from a critical clinical incident, where a patient left the emergency department (unknown to the staff) and died by suicide (see Quotation 4).

Quotation 4: Organisational learning (Characteristic 9, Figure 1 )

I mean certainly that change really changed our practice, but that actually had a national effect because that mental illness triage tool has now been accepted as the National Mental Illness Triage Tool. So other departments across Ireland have taken it on. So, it's not changed only our practice … (Source: Clinician).

### Balmiest hierarchical accountability-attention to culpability and blame

The fourth theme, “attention to culpability and blame”, reveals four characteristics (see Figure 1 , 23–26). Surprisingly, we find little attention given to culpability and blame. Interviewees share a sense of “attention to culpability and blame” in only a few critical clinical narratives. These findings resonate with the just culture context interviewees refer to in earlier quotations.

Most interviewees (28) describe a positive experience in responding to critical clinical incidents, where the practice of accountability is good. However, a few feel that they may be blamed and adopt a defensive approach. Media pressure features in some interviews from a blamist (I.E. THE PRACTICE OF BLAMING OTHERS) perspective. Some interviewees (11) speak about being conscious of and/or impacted by the media’s negative pressures, calling for justification. A sense of the media pressure created is seen in Quotation 5 and Quotation 6.

Quotation 5: Influence of media pressure on blame culture (i) (Characteristic 23, Figure 1 )

We certainly should not take the idea of accountability from the media. I think we should base it not on the idea which is, “off with their head” (Source: Clinician, Manager, Governor)

Quotation 6: Influence of media pressure on blame culture (ii) (Characteristic 23, Figure 1 )

I just think it became a bit of a monster. I think it became a bit insensitive. I think there was a determination to blame. That's my personal view, you know (Source: Clinician, Manager).

The media tends to suggest that healthcare hides errors (e.g. Blackwell, 2015 ; Keane, 2017 ). In high-consequence industries, the many managers’ desire to “hold someone accountable” for errors remains a barrier to advancing meaningful safety agendas ( McCall and Pruchnicki, 2017 , p. 143). Our research points to clinicians, managers and governors realising this not only by taking a more “open” approach to the practice of accountability but also by acknowledging and seeking to mitigate external agencies’ drive to be blamist.

Interviewees (6) also speak about legal pressures (e.g. Quotation 7). A small number of interviewees (5) speak about feelings of being isolated, left on their own or left to themselves to address the problem. Fewer interviewees (4) speak about experiencing or observing a drive to find someone to blame when something is perceived to go wrong. Quotation 8 reflects the “blame game” and attempts towards “blame shifting” ( Pellinen *et al.* , 2018 ).

Quotation 7: Legal pressures (Characteristic 24, Figure 1 )

I have to agree that, in that instance, the legal system really did not support clinical practice and open disclosure (Source: Manager Governor).

Quotation 8: Seeking who is to blame (i) (Characteristic 26, Figure 1 )

Whereas now it's who can we blame, who can we hold accountable and accountability nowadays is who do we blame, as before I firmly believe accountability was about who can make the difference, how can we change something … (Source: Manager)

In contrast, in Quotation 9, a clinician manager talks about a review of an incident where a patient fell out of bed during the night and subsequently died where no one was scapegoated.

Quotation 9: Seeking who is to blame (ii) (Characteristic 26, Figure 1 )

[…] I thought that that was refreshing in so much as there was no question of this being brushed under the carpet but there was also no real question of somebody being scapegoated (Source: Clinician, Manager).

Using critical clinical incidents to learn and make changes proactively in practice is the main driver of accountability. Internal hospital accountability does not operate in a blamist manner. However, interviewees perceive external bodies to be determined to seek someone to blame.

Our study reveals interviewees’ awareness of the dangers of an imposing or demanding approach to accountability. For example, a manager, who is also a board member, illustrates this when talking about an incident where there was a breakdown in communication and handover between clinicians (see Quotation 10).

Quotation 10: Embracing failure as an opportunity for learning (Characteristic 19, Figure 1 )

… And there was not a blame culture – it was like, how did it happen, why did it happen, how can we stop this happening again, so because if you start having a blame culture people are not going to start reporting incidents and events, and I think that's where the whole open disclosure comes along, you go hands-up, we've made a mistake here. (Source: Manager, Governor)

Surprisingly, there was little discussion about money, cost or potential financial losses from critical clinical incidents. We find financial considerations do not dominate clinical ones. When confronted by adverse medical outcomes, clinicians and most managers are not concerned with financial implications. The bottom-line for most interviewees is supporting patients and staff affected and making changes in practice to increase safety and quality of services. We find learning from the incident or making sure it does not recur (a form of accountability), rather than budgets, is the main concern in interviewees’ responses to critical clinical incidents. Only two interviewees (both members of the hospital board) mentioned finance but only in passing.

### The emergent concept “grounded accountability”

Our research identifies an emergent concept, “grounded accountability”. Quotation 11 illustrates a sense of what “grounded accountability” means, when a manager, who is also a governor, describes a meeting with members of a family who had a serious complaint about their brother’s care when in hospital.

Quotation 11: Grounded accountability

I suppose the piece that I feel most strongly about is the fact that if the Board of Directors are at a distance from staff and if there is any element of “them and us”, then I think that is very sad and I would be very disappointed and upset if I thought that existed because I would see the Board of Directors as being there for the sole purpose of helping the staff to achieve what is the best practice for the patient and there is one mantra that we have – “The patient always comes first”. But in order to achieve what is best for the patient and in putting the patient first, if the staff are not equipped to do it, if they have not the environment to do it, if they do not feel free to present their issues, if they have issues, then that will not be achieved (Source: Manager, Governor).

When managers and governors care for staff and listen to their concerns and suggestions, front-line staff describe feeling safe and more willing to tackle and change practices (see Quotation 4). Most interviewees (22) are not so much concerned about their reputation. Instead, interviewees are concerned for their colleagues and the hospital’s reputation. Therefore, clinicians are more willing to take risks (reporting rather than concealing errors/mistakes) because of a feeling that the hospital has “got my back”, as understood by a non-executive board member in Quotation 12.

Quotation 12: Ensuring clinicians feel safe

People have to know that if they've messed up, you know, does the organisation have their back?. (Source: Governor)

### Co-construction of “grounded accountability”

We reveal that clinicians, managers and governors co-construct “grounded accountability”, which resonates with the prior literature ( Checkland *et al.* , 2004 ). Grounded engagement (Theme 2) by managers/governors, along with front-line staff’s felt accountability (Theme 1), supports a culture of openness (Theme 3) to achieve the ongoing process of “grounded accountability”. Thus, the three themes lead to co-constructed accountability, which is “grounded”. Nurtured by managers and governors, front-line staffs’ sensitivity to a felt accountability shaped by their collective, unconsciously learned responses and a repertoire of practices enable staff to respond in an open, accountable way to critical clinical incidents.

**Figure 2:**
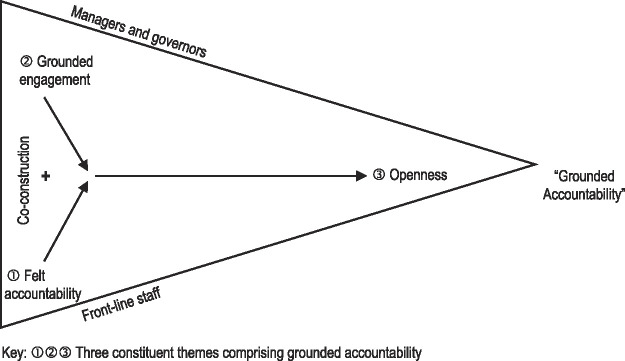
Conditions necessary for co-construction of “grounded accountability”

We present the dynamic interrelationships between the concept’s three constituent themes in Figure 2 . Three conditions are necessary for “grounded accountability”: (1) Attention by front-line staff to a felt accountability, together with (2) attention by managers and governors to grounded engagement. These two conditions then support the third condition, namely, (3) an overall attention to openness, which, in turn, creates the conditions for “grounded accountability”. Attention to openness depends on both attention to grounded engagement and attention to felt accountability. Consequently, “grounded accountability” will not occur if only one element is present.

Grounded accountability does not lead to co-constructed accountability. Rather, co-construction is a precursor to grounded accountability. This co-construction between front-line staff and managers/governors makes the achievement of a “grounded accountability” possible. Collaboration between both groups is necessary for co-construction to take place. Co-construction of accountability is the antithesis of the more traditional “them and us” stance between clinicians and managers/governors. Without this co-construction, there are potential obstacles to delivering “grounded accountability” (e.g. an unwillingness to share patients’ medical records with them or to extending an apology when something goes wrong).

### The practice of accountability revealed by interviewees

Rather than accountability being “giving and demanding of reasons for conduct” ( Roberts and Scapens, 1985 ), we find accountability in our study being an environment of “the giving and enquiry about reasons for action” – “enquiry about” rather than “demanding of” and “reasons for action” rather than “reasons for conduct”. Our findings highlight voluntary disclosure of events leading to the enquiries that are reviews-for-learning rather than adversarial investigations. Interviewees describe multiple contexts where they gave reasons for conduct ( Table 2 ). Account giving takes the form of face-to-face verbal reports (101), written incident reports (51) and formal reports reviewing the critical clinical incident (where one was undertaken).

We find clinicians instinctively know how to demonstrate accountability, confirm to the people concerned that the matter has been reviewed, establish explanations and the chronology/facts of the events, and identify and implement ameliorating actions/improvements. In Quotation 13, the review would not have taken place except that the clinician sought and ensured it happened. Managers had previously decided an investigation was not required.

Quotation 13: Demonstrating accountability

… so, it's vitally important for me that an investigation took place into this so that when I see these patients again in three months that I can hand them the document and say: this is what's changed, this is what's changed on the computer, this is what's changed in how we do it. It was not OK for me to just be able to say to them, oh we took care of it … (Source: Clinician)

## Conclusion

This research takes a novel theoretical perspective by building a new concept through a fusion from two scholarly sources: (1) accountability (Roberts, O'Dwyer, and colleagues) and (2) management (Mintzberg), together with insights from practices in hospital settings. The new emergent concept “grounded accountability” is thus theoretically and practically anchored. In a potentially life-and-death, high-consequence hospital setting, a more nuanced, subtle and sensitive approach may be more appropriate than traditional considerations of accountability. Our research provides a new way of looking at accountability. We introduce the emergent concept of “grounded accountability”, with its 22 characteristics, a potentially useful concept, with practical application. We offer this emergent concept “grounded accountability” as a more intelligent accountability, with the potential of transforming the practice of accountability.

We find multiple accountability practices in the study hospitals: (1) proactively taking responsibility (reporting internally and externally), (2) reviews-for-learning, (3) providing face-to-face explanations, (4) making changes in practice and (5) disciplinary action involving sanctions in a small number of cases. Most interviewees in the study hospitals are open with each other and patients about performance, learn from mistakes (without apportioning blame) and take ameliorating action to improve practices. Our research identifies a strong focus on the processes of giving account and speaking up during abnormal events. This openness emanates from policies and procedures focused on creating nurturing cultures, treating staff and patients compassionately and fairly when systems fail and errors occur. Our study suggests an overriding ethos amongst managers and governors of going beyond first impressions to understand the deeper and more complicated story through clinicians’ and patients’ narratives. This culture enables clinicians, managers and patients/families to play a role in preventing future errors – resulting in “grounded” accountability emerging from the front-line.

Hierarchical accountability structures tend to be focused on efficiency and performance rather than the quality of care. In this paper, we advance understanding of accountability in a healthcare context by attending to the co-construction of accountability from the ground upwards, focusing on individual-level rather than organisational-level accountability. Such horizontal accountability approaches are suitable in autonomous decision-making contexts, such as exercised by professionals within a network or peer-group structure ( Vrangbæk and Byrkjeflot, 2016 ).

Some important unanswered questions arise form this research for further research. The 22 characteristics of “grounded accountability” warrant further exploration to determine whether there is a hierarchy of importance amongst them and determine if there is any overlap and the nature of that overlap. There is also an opportunity to explore the relationship between culture and accountability in further studies. Reason (1997 – as cited in Bitar *et al.* , 2018 ) recommends a just culture decision tree of diminishing culpability. Similarly, a next step in developing our grounded accountability concept might be to more formally map the system processes implied by the concept.

The study has some limitations. While we pay careful attention to interviewees’ reflections on patient and family perspectives and experiences, our study does not include patient/family experiences of the critical clinical incidents. While the context for this study is individual hospitals, there is growing recognition that for complex interdependent healthcare provision (between community and hospitals), viewing accountability through the lens of “service-as-a-system”, rather than an organisational lens, could provide insights into user/provider interactions ( Virtanen *et al.* , 2018 ). The “service-as-a-system” lens could provide a new perspective from which to view accountability. Finally, interviewee experiences are limited to the study hospitals and other hospitals where they have worked and therefore may not represent hospitals in general.

Our perspective is that the practice of accountability is more of a process than an outcome. The practice of accountability is not a one-off compliance event but is ongoing. It is continuous, applies all the time and functions at all levels in an organisation. This form of accountability is dynamic, continuously in dialogue with on-the-ground front-line staff in a reflexive manner. In complex settings, individual and administrative responsibilities are intrinsically linked, the implication for practice being a collective responsibility for high standards and quality. The Irish approach to critical clinical incidents, contained in the “Incident Management Framework” ( Health Service Executive, 2020 ) and earlier versions, is based on international best practice and is similar to that used in many other countries. Therefore, we believe the insights from our study apply to acute hospitals beyond the Irish context for our research. However, the structure, processes and cultures may vary between hospitals in different geographic areas. The applicability of our grounded accountability concept in different settings is a matter for future research. Nevertheless, grounded accountability provides a way forward, as it brings front-line staff, managers and governors (who operate the system) and a person orientation together, making sure that one does not apply in isolation to the other.

## Figures and Tables

**Table 1:** Interviewees

Interviewee position	Interviewee category	Hospital A	Hospital B	Clinician Note(s)	Administrator	Total
		No	No	No	No	No
Health and social care professional	Clinician	1	1	2	–	2
Advanced nurse practitioner	Clinician	1	1	2	–	2
Hospital medical consultant	Clinician	5	4	9	–	9
Quality manager*	Clinician/Manager	1	1	2	–	2
Risk manager*	Manager	1	–	–	1	1
Chief operating officer*	Clinician/Manager	1	1	2	–	2
Director of human resources*	Manager	1	1	–	2	2
Quality and safety committee: chair*	Clinician/Manager	1	1	2	–	2
Director of nursing*	Clinician/Manager/Governor	1	1	2	–	2
Clinical director/medical director*	Clinician/Manager/Governor	1	1	2	–	2
Director strategy/mission*	Manager/Governor	1	1	1	1	2
Director of finance*	Manager/Governor	1	1	–	2	2
Chief executive officer*	Clinician/Manager/Governor	1	1	1	1	2
Board committee: chair/member*	Clinician/Governor	1	1	2	–	2
Board: non-executive director	Clinician/Governor	2	3	1	4	5
Board: chair*	Governor	1	1	–	2	2
Total		21	20	28	13	41

**Note(s):** Some clinicians also hold governor/manager roles simultaneously

Key: *Invited to participate because of position held

**Table 2:** Format of account-giving where interviewees report providing reasons for their conduct

Format of report following critical clinical incident (CCI)		Hospital A *(21 CCIs)	Hospital B *(31 CCIs)	Total
		No	No	No
In person	Internal to manager/governor	22	25	47
	To patient/family members(s)	19	19	38
	To external agencies, e.g. coroner, professional regulator, insurer, police	6	10	16
	*Subtotal*	*47*	*54*	101
In writing	Internal to manager/governor	18	20	38
	To patient/family members(s)	2	6	8
	To external agencies, e.g. insurer, coroner, professional regulator, ombudsman, police, media	–	5	5
	*Subtotal*	*20*	*31*	51
Total		67	85	152

**Note(s):** Key: *Interviewees from Hospital A and Hospital B describe the same incident i.e. 58 descriptions of 50(52) CCIs

## Notes

1.
Scobie *et al* . (2021) also use the term “grounded accountability”, derived from the indigenous New Zealand Âāori term “whakapapa” (genealogy). By grounded accountability, Scobie *et al* . (2021 , p. 1) mean “an accountability driven by reciprocal relationships and responsibilities grounded in kinship, place and intergenerationality”.

2. The research received clearance from University College Dublin as follows: Research Ethics Exemption Reference Number (REERN) is: LS-E-15-127-Flynn-Brennan (confirmation received 22nd October 2015).
